# Supplementary material for: Recent upper Arctic Ocean warming expedited by summertime atmospheric processes
Source: Nat Commun. 2022 Jan 18;13:362. doi: 10.1038/s41467-022-28047-8 (PMC8766491; doi:10.1038/s41467-022-28047-8)
Supplement: Supplementary file 1 — Supplementary Information [file 41467_2022_28047_MOESM1_ESM.pdf]

Supplementary material for

Recent upper Arctic Ocean warming expedited by summertime atmospheric processes

Zhe Li[1], Qinghua Ding[1], Michael Steele[2], Axel Schweiger[2]

1. Department of Geography, and Earth Research Institute, University of California, Santa Barbara, Santa Barbara, California, USA
2. Polar Science Center, Applied Physics Laboratory, University of Washington, Seattle, Washington, USA

**Corresponding author address:* Dr. Qinghua Ding, Department of Geography, and Earth Research Institute, University of California, Santa Barbara, Santa Barbara, California, USA.

Email: Qinghua@ucsb.edu

Supplementary figures for Li et al. “Recent upper Arctic Ocean warming expedited by …”

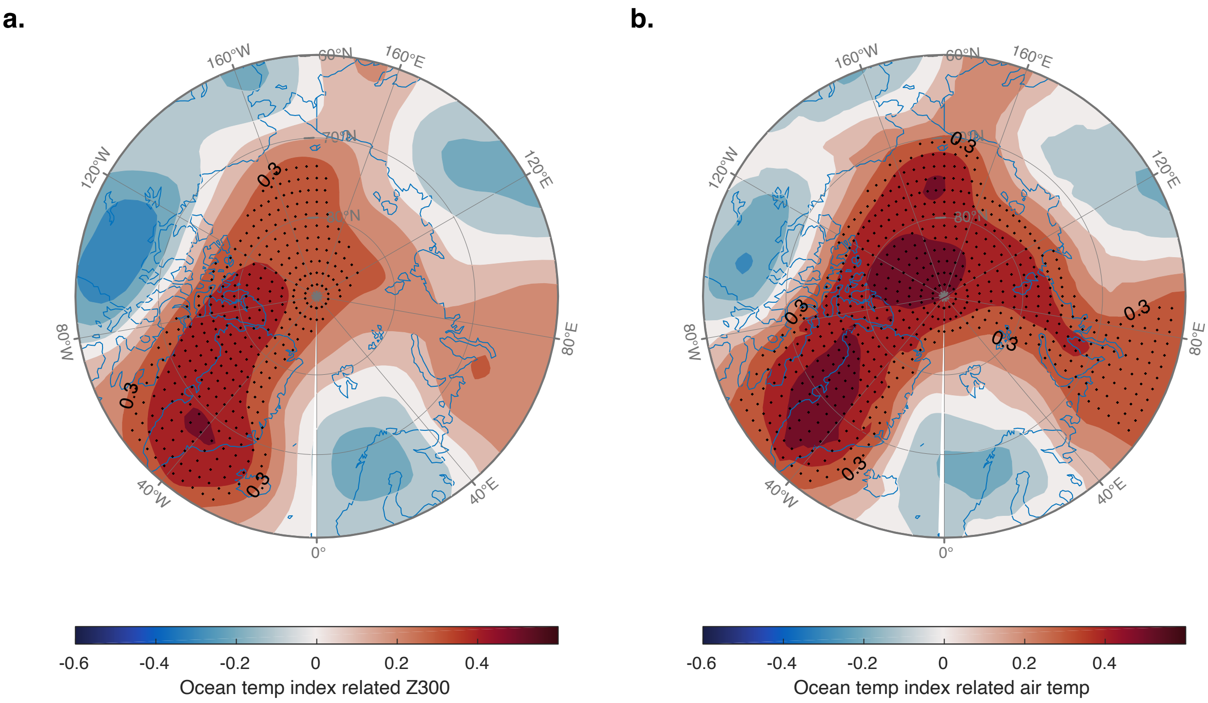

Supplementary Fig. 1. Correlation of domain-average SON upper ocean temperature in **Fig. 1a** with the spatial fields of JJA Z300 in **a** and JJA tropospheric (surface to 300 hPa average) air temperature in **b** for the period 1979 – 2018. All linear trends are removed in calculating the correlations. Black stippling in all plots indicates statistically significant correlations at the 95% confidence level.

To investigate whether the links between atmospheric warming and ocean are tied to fundamental modes of variability with a spatially coherent structure, we performed a maximum covariance analysis (MCA) between the JJA atmospheric circulation and SON upper (0 - 50 m average) ocean temperature in the Arctic. Supplementary Fig. 2a-c show results using detrended variables. The patterns and correlations are similar to those using raw data (not shown). The leading mode of MCA analysis accounts for the majority of covariance (36%, see Methods) and dominates the linkage of the two fields. The ocean temperature pattern of MCA1 exhibits increased temperature mostly in the PPSS and is closely coupled with a Z300 pattern characterized by a prominent high pressure over northeastern Canada and Greenland, which has been suggested by Ding et al. [36] to be a critical driver of sea ice variability in the past decades (Supplementary Fig. 2a&b). The MCA1 time series of the two patterns are highly correlated (r = 0.74) and are almost identical to the time evolution of detrended observed upper Arctic domain-average ocean temperature in SON (Supplementary Fig. 2c). The Z300 and ocean temperature patterns revealed by the leading MCA mode feature a striking resemblance with the observed trend patterns in JJA Z300 and SON upper ocean temperature fields (Supplementary Fig. 2a&b & Fig. 1c&d), suggesting that there exists a physical link between changes in observed upper ocean temperature and the atmospheric circulation characterized by the Z300 pattern. Since the MCA analysis is performed on detrended data, the close match between the observed trends and the MCA patterns suggests that the observed trend to a substantial degree arises from internal variability rather than secular trends.

To further illustrate the association between atmospheric and oceanic temperature, we calculate the lead-lag correlation between the MCA1 JJA Z300 time series with domain-average air temperature within the Arctic for each month and layer (Supplementary Fig. 2d&e). Prominent ocean temperature rise succeeding the JJA atmospheric warming starts to appear in early summer near the surface and then exhibits a strong downward intrusion into lower layers to ~ 50 m until the following fall and even winter (Supplementary Fig. 2e). This lead-lag correlation pattern looks very similar to the temporal evolution pattern associated with the observed JJA tropospheric air temperature (Fig. 1h) and long-term trend of upper ocean temperature in the Arctic Ocean (Fig. 1f), suggesting the contribution of recent summertime atmospheric warming to the following upper Arctic Ocean warming in SON.

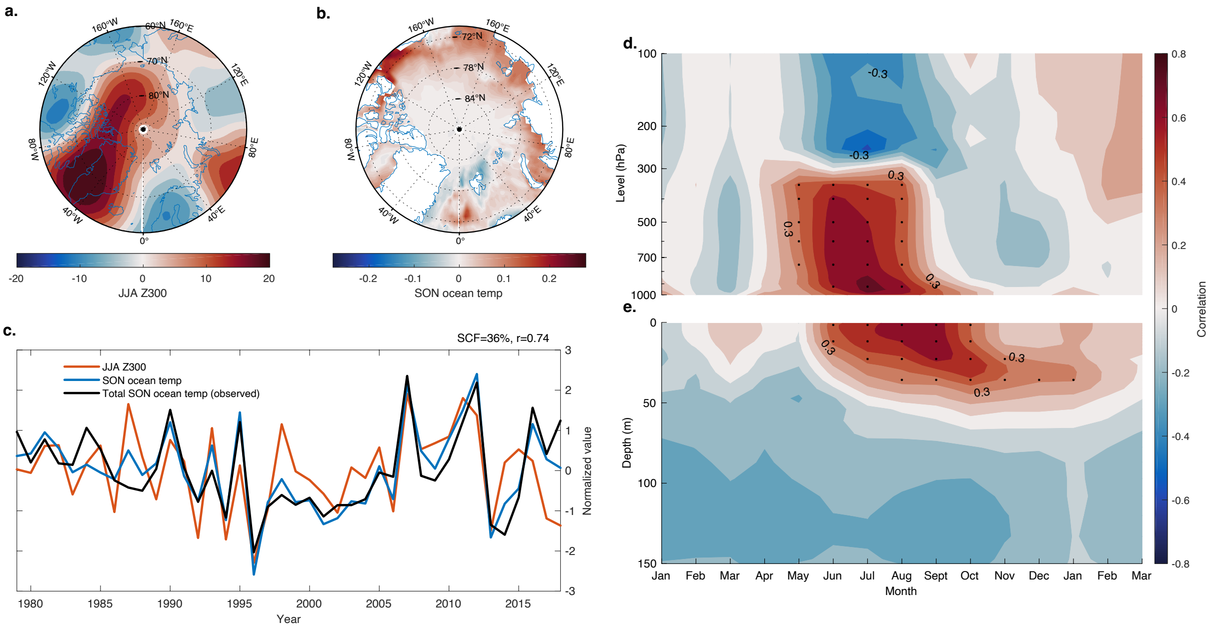

Supplementary Fig. 2. a-c The leading patterns of covariability and their time series from a MCA of JJA Northern Hemisphere high-latitude (60° – 90°N) Z300 and SON upper (0 – 50 m average) ocean temperature in the Arctic (70° – 90°N) for the period 1979 – 2018, where all fields are detrended. The patterns of Z300 and ocean temperature that accompany the first MCA mode are shown in **a** and **b**, respectively. The normalized time series of the MCA1 JJA Z300 (red line) and MCA1 SON ocean temperature (blue line) patterns, and total SON domain-average upper ocean temperature from the reanalysis (black line) are shown in **c**. The squared covariance fraction (SCF) showed in **c**. indicates that the first mode accounts for 36% of the covariance, and the correlation between the time series of JJA Z300 and SON ocean temperature mode is 0.74. **d&e** Correlation of MCA1 JJA Z300 time series (red line in **c**) with domain-average air temperature in **d**, and with domain-average ocean temperature in **e**, for each month (the last three months: Jan-Feb-Mar are the months in the next year) and layer (atmosphere: from 1000 – 100 hPa; ocean: from 0 – 150 m) for the period 1979 – 2018. All linear trends are removed in calculating the correlations in **d&e**. Black stippling in all plots indicates statistically significant correlations at the 95% confidence level.

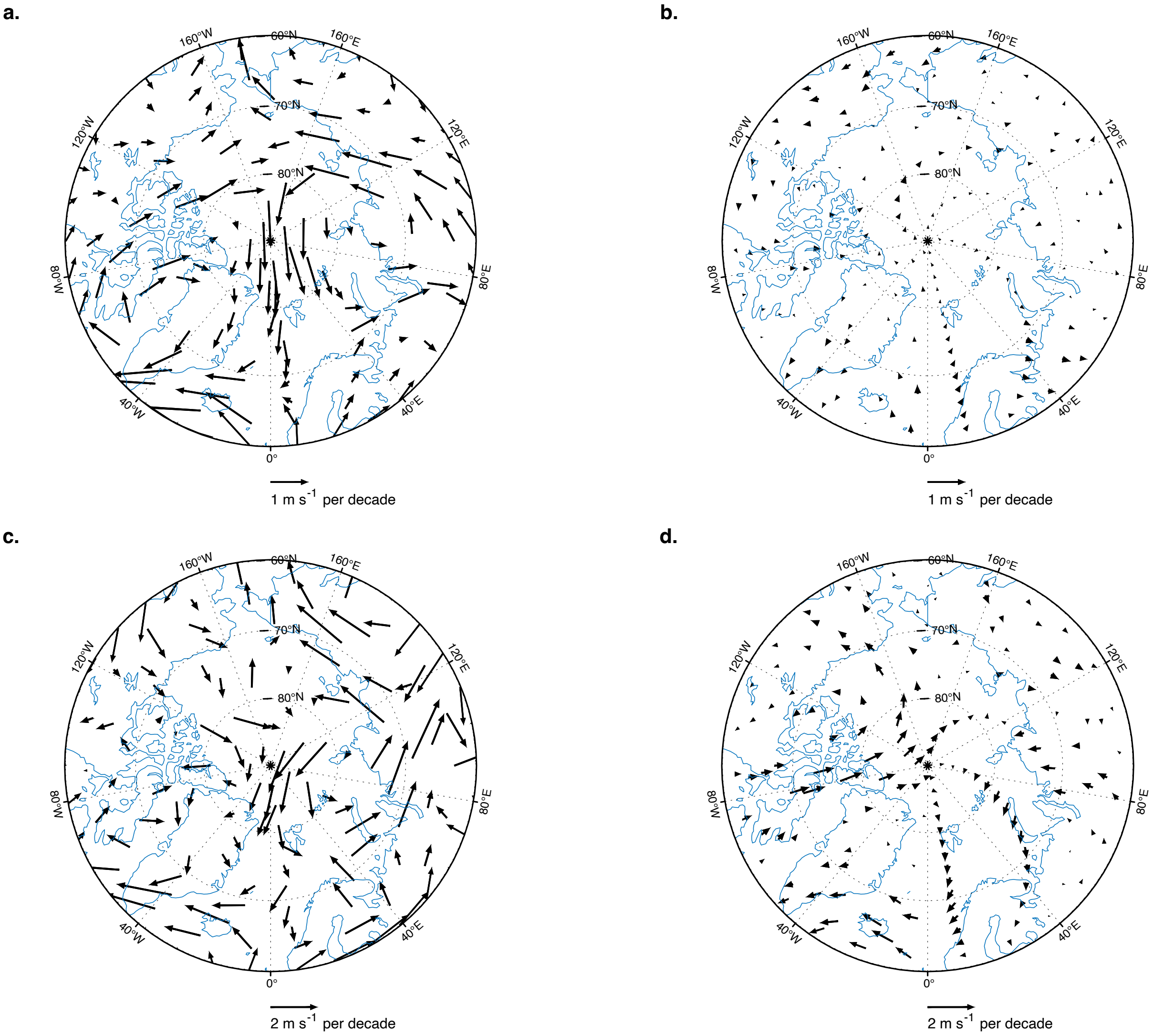

Supplementary Fig. 3. Linear trends of JJA 300hPa wind (m s^{-1} per decade) from the ERA5 reanalysis for the period 1979 - 2018 in **a** and the period 2000 - 2018 in **c** and from the 40-member ensemble mean of CESM-LEN for the period 1979 - 2018 in **b** and the period 2000 - 2018 in **d**.

Supplementary figures for Li et al. "Recent upper Arctic Ocean warming expedited by …"

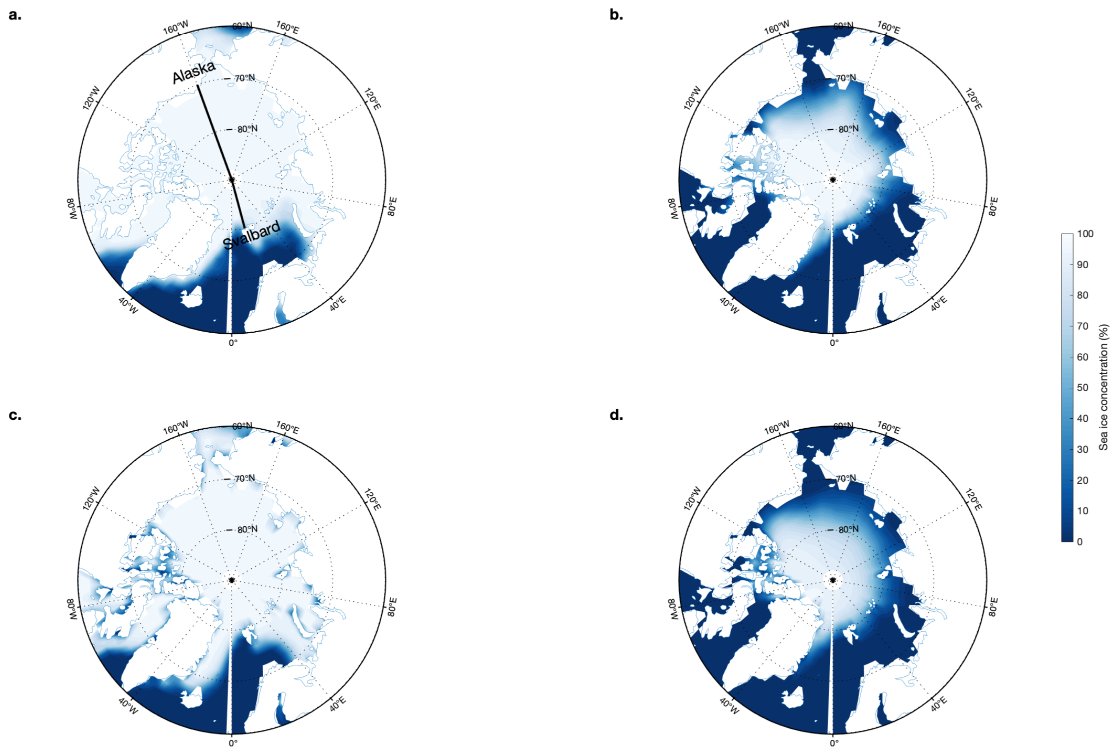

Supplementary Fig. 4. a&c Climatology of March sea ice concentration from the NSIDC Nimbus-7 SMMR and DMSP SSM/I-SSMIS passive microwave monthly sea-ice product version 1 in **a** and from the ensemble average of the five wind nudging experiments in **c** for the period 1979 - 2018. **b&d** Climatology of September sea ice concentration from the NSIDC Nimbus-7 SMMR and DMSP SSM/I-SSMIS passive microwave monthly sea-ice product version 1 in **b** and from the ensemble average of the five wind nudging experiments in **d** for the period 1979 - 2018. The solid black line in **a** from Alaska to Svalbard is used in **Supplementary Fig. 5** for the vertical cross section plots.

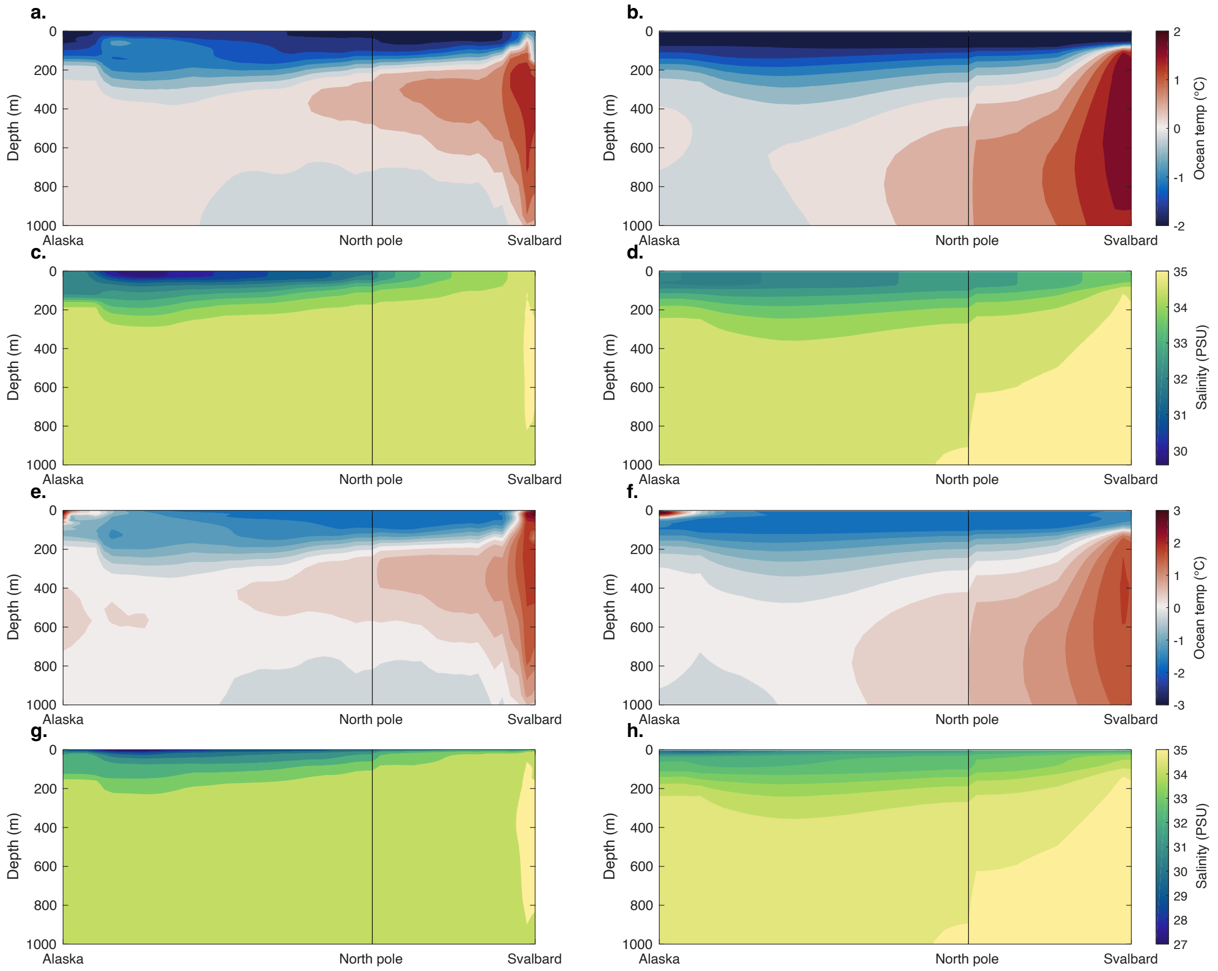

Supplementary Fig. 5. a&b Climatology of February ocean temperature over the years 1979 – 2018 for a vertical cross section Alaska-North Pole-Svalbard over 0 – 1000 m depth from the ORAS5 reanalysis in **a** and from the ensemble average of the five wind nudging experiments in **b. c&d** Same as **a&b** but for salinity**. e-h** Same as **a-d**, but for mean August.

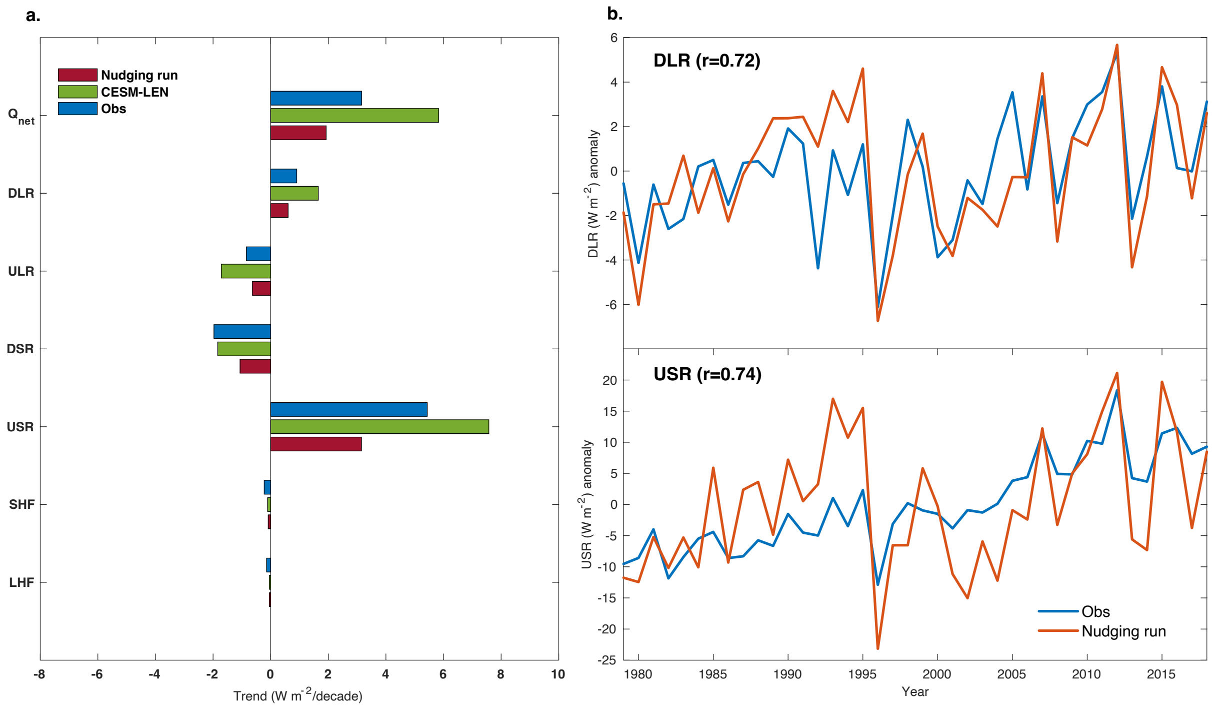

Supplementary Fig. 6. a Linear trends (W m^{-2} per decade) of JJA Arctic domain-averages of net heat flux (Q_{net}), DLR, ULR, DSR, USR, SHF, and LHF in the ensemble average of the five wind nudging experiments (red bars), the ensemble average of CESM-LEN 40 members (green bars), and the ERA5 reanalysis (blue bars) (1979 - 2018), and all radiative flux variables are positive downward. **b** JJA Arctic domain-average DLR and USR anomalies (unit: W m^{-2}) in the ensemble average of the five wind nudging experiments (red line) and the ERA5 reanalysis (blue line).

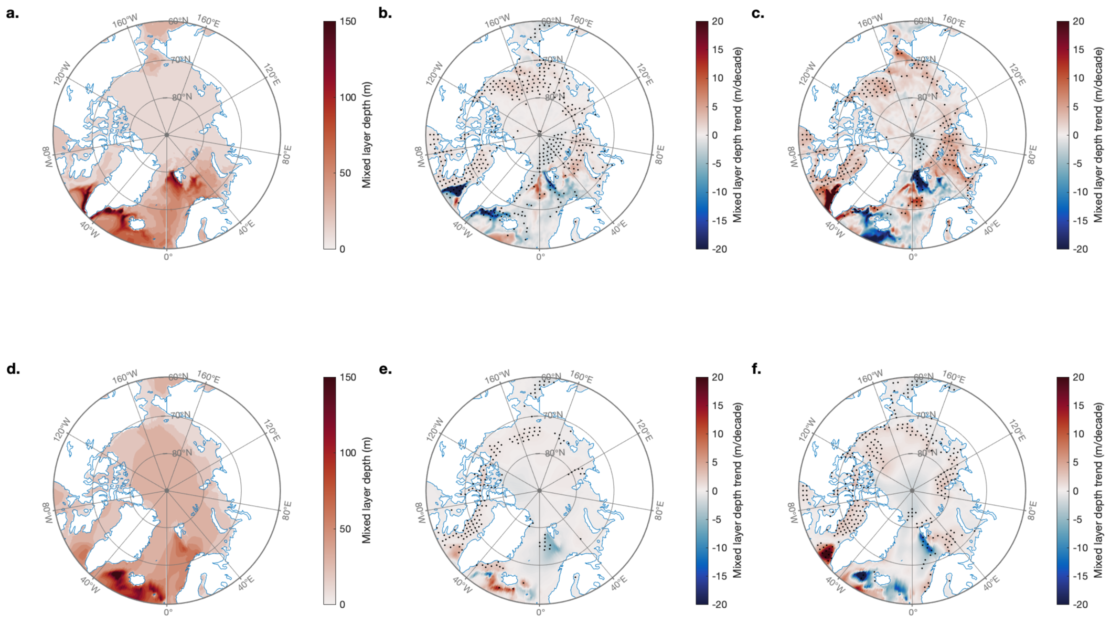

Supplementary Fig. 7. **a** Climatology (m) of the mixed layer depth in SON from the ORAS5 reanalysis for the period 1979 – 2018. **b-c** Linear trend (m per decade) of the mixed layer depth in SON for the period 1979 – 2018 in **b**, and for the period 2000 – 2018 in **c** from the ORAS5 reanalysis. **d-f** Same as **a-c**, but from the ensemble average of the five wind nudging experiments. Black stippling in all plots indicates statistically significant trends at the 95% confidence level.

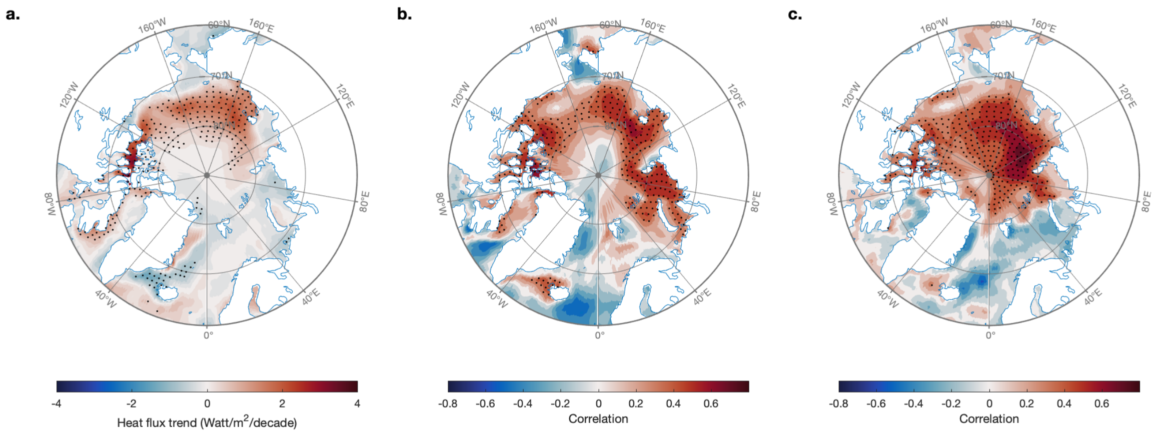

Supplementary Fig. 8. **a** Linear trend (W m^{-2} per decade) of solar shortwave heat flux in the ocean boundary layer (Q_{short_bl}) field in SON from the ensemble average of the five wind nudging experiments for the period 1979 – 2018. **b-c** Correlation of JJA domain-average net heat flux (Q_{net}) with Q_{short_bl} field in JJA in **b**, and in SON in **c** from the ensemble average of the five wind nudging experiments for the period 1979 – 2018. Black stippling in all plots indicates statistically significant correlations or trends at the 95% confidence level.

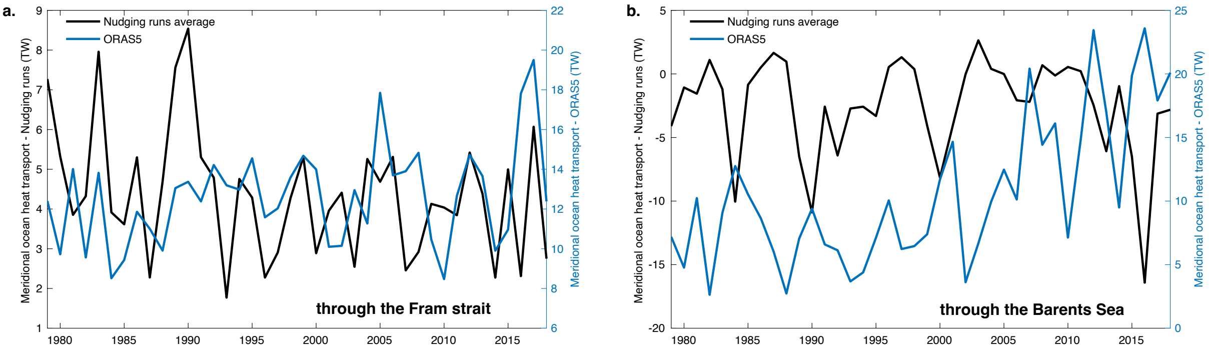

Supplementary Fig. 9. SON POHT (TW) within upper 50 m from 1979 to 2018 through the Fram Strait in **a,** and through the Barents Sea in **b** from the ensemble average of the five wind nudging experiments (black line) and the ORAS5 reanalysis (blue line).

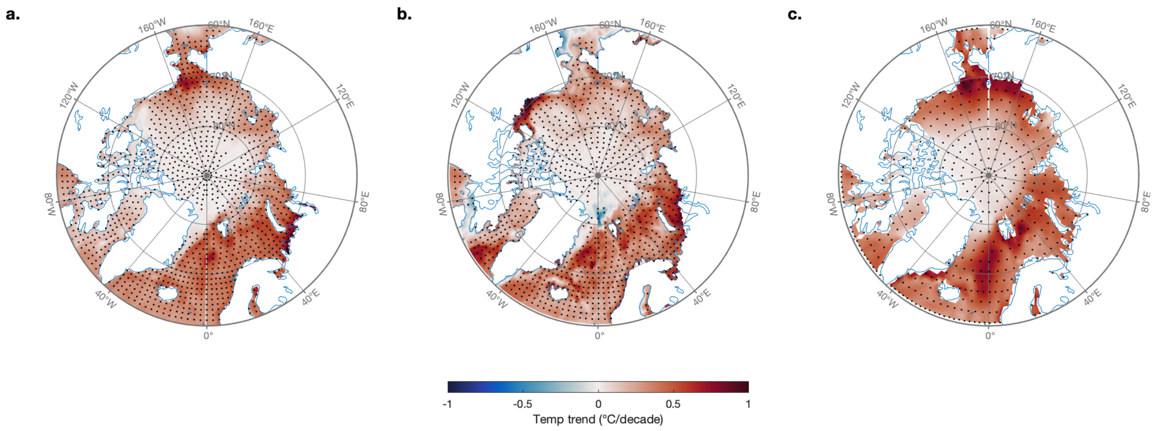

Supplementary Fig. 10. Linear trend (°C per decade) of SON upper ocean temperature using three different reanalysis data (ORAS5 in **a**, SODA3.4.2 in **b**, and GECCO3 in **c**) (1979 – 2018). SODA3.4.2 only provides data from 1980 to 2016, which is different from the other two reanalyses. Black stippling in all plots indicates statistically significant trends at the 95% confidence level.

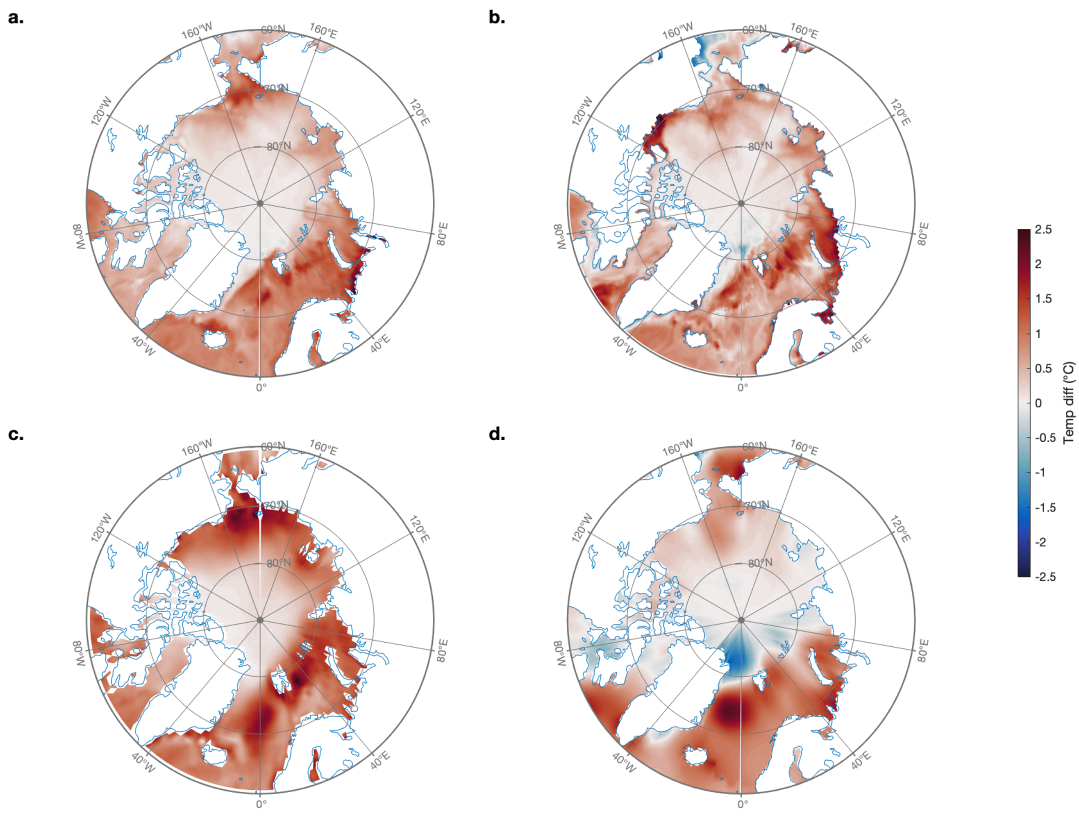

Supplementary Fig. 11. Differences of SON upper ocean temperature between the periods 1985 – 1994 mean and 2005 – 2017 mean using three different reanalyses (ORAS5 in **a**, SODA3.4.2 in **b**, and GECCO3 in **c**) and observation data (WOA18 in **d**). SODA3.4.2 only provides data from 1980 to 2016, which excludes the data of year 2017 to calculate temperature difference.

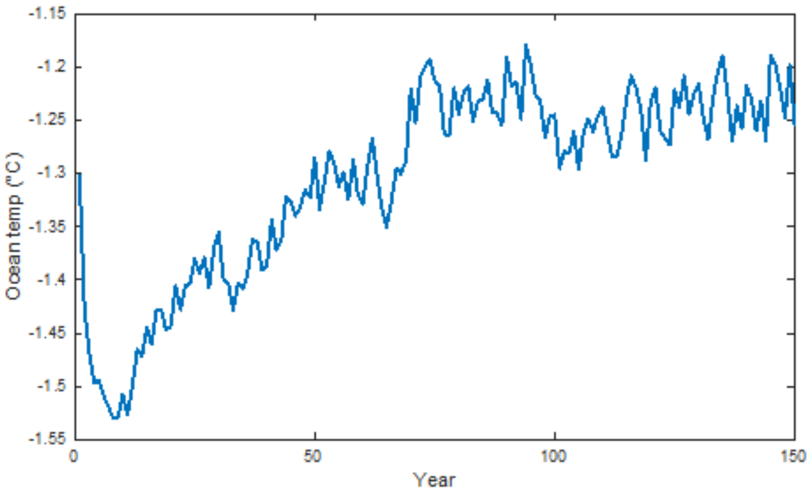

Supplementary Fig. 12. The Arctic Ocean domain-average upper (0 – 50 m average) ocean temperature (°C) in SON during 150-yr spin-up perpetual run.

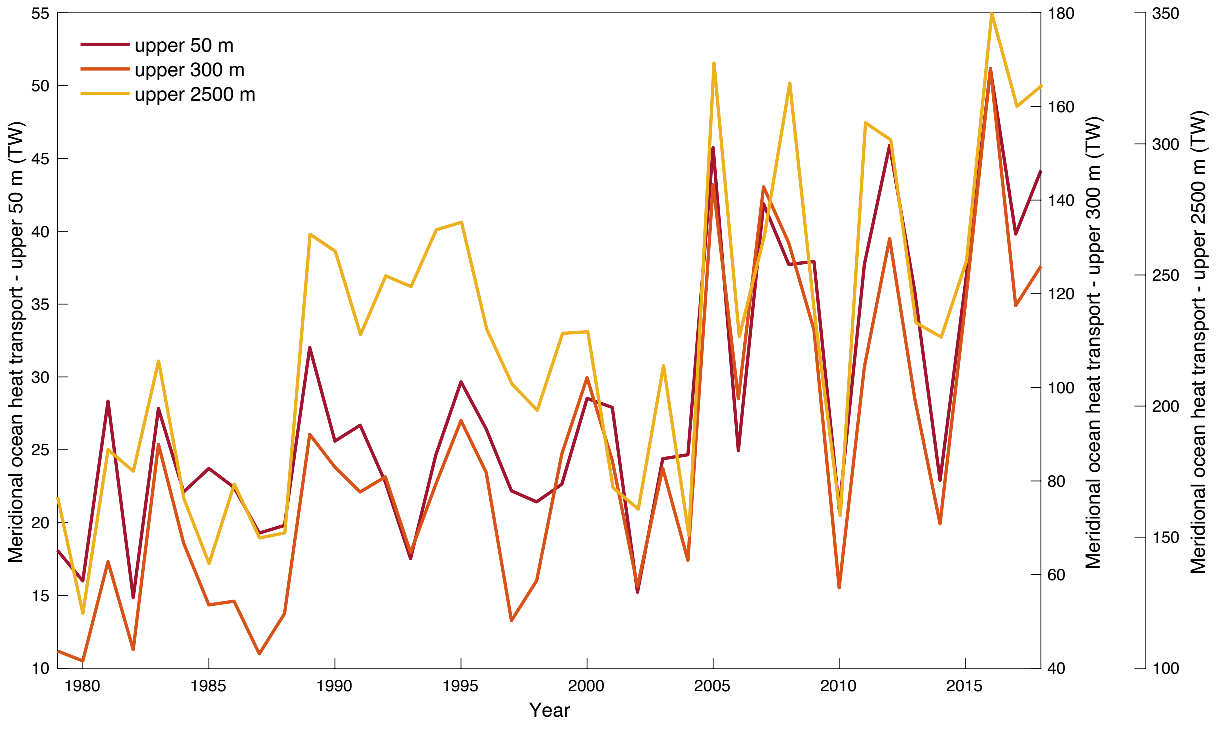

Supplementary Fig. 13. SON POHT (TW) through the Atlantic Gate within the upper 50 m, 300 m, and 2500 m from 1979 to 2018 using the ORAS5 reanalysis.

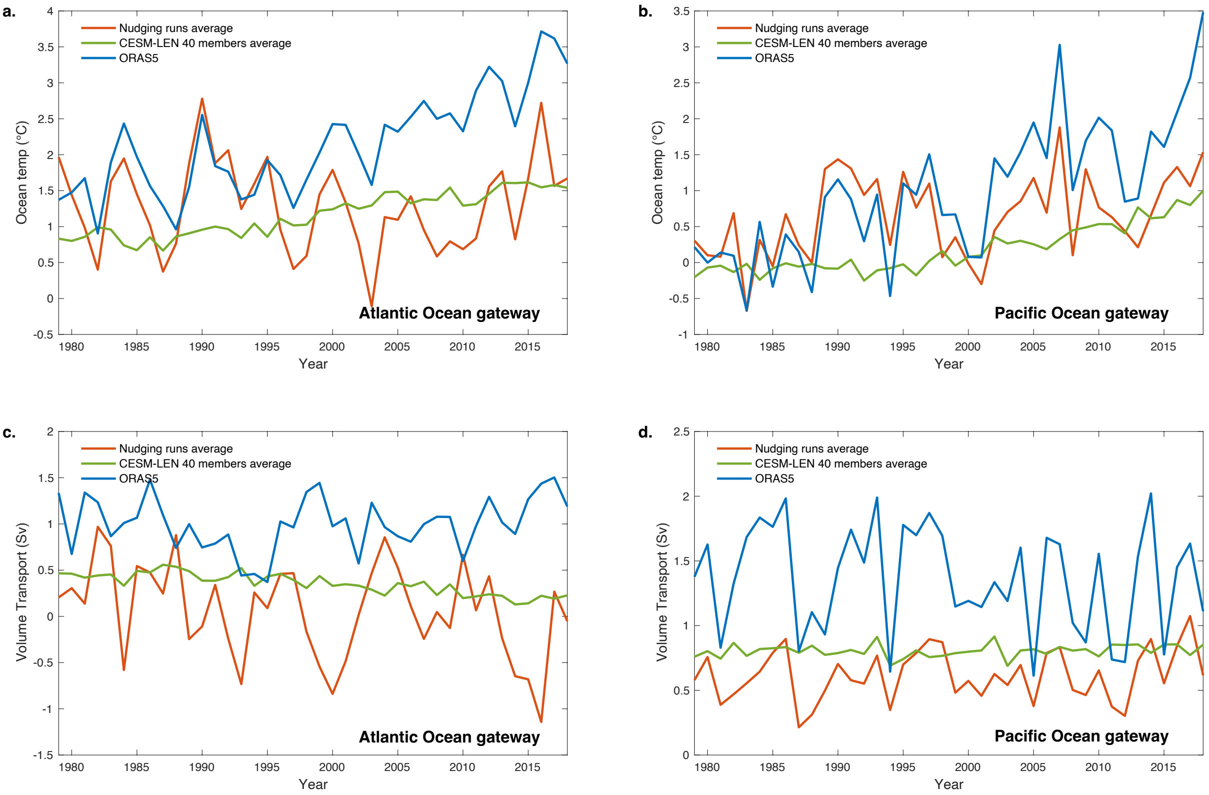

Supplementary Fig. 14. **a&b** SON cross section-average upper (0 – 50 m average) ocean temperature (°C) along the Atlantic Ocean gateway in **a**, and along the Pacific Ocean gateway in **b** from the ensemble average of the five wind nudging experiments (red line), the CESM-LEN 40 members average (green line), and the ORAS5 reanalysis (blue line) for the period 1979 – 2018. **c&d** Same as **a&b** but for the poleward volume transport (Sv) in the upper 50 m in SON.
